# 套细胞淋巴瘤诊断与治疗中国指南（2022年版）

**DOI:** 10.3760/cma.j.issn.0253-2727.2022.07.001

**Published:** 2022-07

**Authors:** 中国抗癌协会血液肿瘤专业委员会, 中华医学会血液学分会, 中国临床肿瘤学会淋巴瘤专家委员会

套细胞淋巴瘤（mantle cell lymphoma, MCL）是一种起源于成熟B细胞的非霍奇金淋巴瘤亚类，占非霍奇金淋巴瘤（NHL）的6％～8％[1]。自《套细胞淋巴瘤诊断与治疗中国专家共识（2016年版）》发布以来[2]，极大地促进了我国医务工作者对该病的认识，并规范了临床诊疗。近年来，MCL的基础转化研究与临床治疗均取得重大进展。为进一步提高我国医药工作者对该病的认识，推广规范诊疗，中国抗癌协会血液肿瘤专业委员会、中华医学会血液学分会与中国临床肿瘤学会淋巴瘤专家委员会共同组织国内相关专家经过多次讨论，制订本版指南。

一、定义

MCL是一种具有特定免疫表型和重现性遗传学异常的小至中等大小、单形性成熟B细胞肿瘤，通常表达CD5和SOX11，95％以上患者伴有CCND1基因重排并导致Cyclin D1蛋白细胞核内高表达；患者以老年男性为主，常侵犯结外部位，兼具侵袭性淋巴瘤疾病进展迅速和惰性淋巴瘤不可治愈的特点[1]。

二、诊断、鉴别诊断、分期和预后

（一）诊断

1. 临床特点：中位发病年龄约60 岁，男女比例为2～4∶1。诊断时80％以上患者处于疾病晚期（Ann Arbor Ⅲ～Ⅳ期），表现为淋巴结肿大、脾肿大及骨髓或外周血受累，其他常见的结外受累部位为胃肠道和韦氏环[3]。

2. 组织形态学特点：MCL多呈弥漫性、结节状或套区型生长。典型的MCL常由形态单一、小到中等大小淋巴细胞构成，核轻度不规则，染色质浓聚、核仁不明显，细胞质较少。部分病例可出现单核样B细胞或浆细胞性分化。病灶微环境中多有滤泡树突细胞增生及T细胞浸润，玻璃样变性小血管增生和上皮样组织细胞增生也很常见。不同病例的核分裂数差异较大。10％～15％的MCL细胞形态呈“侵袭性变型”，侵袭性变型又可分为母细胞变型和多形性变型，瘤细胞体积大，且通常具有较高的增殖活性。这些患者临床侵袭性较高，预后差。

3. 免疫表型特点：肿瘤细胞应为单克隆性成熟B淋巴细胞，典型的免疫表型为CD5、CD19、CD20阳性，CD23和CD200阴性或弱阳性[4]，BCL2、CD43常阳性，强表达sIgM或IgD，CD10和BCL6偶有阳性。免疫组化Cyclin D1核内强阳性是MCL相对特异性的免疫标志，经典型MCL常伴有SOX11阳性。

4. 细胞分子遗传学特点：染色体t（11;14）（q13;q32）导致CCND1基因与免疫球蛋白重链（IGH）基因易位是MCL的遗传学基础，见于95％以上的MCL患者。约5％的MCL患者可无t（11;14）[5]，这些患者中约55％可伴有CCND2基因重排，主要与免疫球蛋白轻链基因发生易位[6]。90％以上的MCL继发其他遗传学异常，包括染色体拷贝数异常和基因突变[7]–[9]。

5. 诊断与分型：

（1）诊断：主要依据典型的组织形态学特征联合成熟B细胞免疫特征，加免疫组化CD5和Cyclin D1核内阳性。对于白血病性非淋巴结型MCL，如肿瘤细胞免疫表型符合典型MCL、常规染色体核型分析或荧光原位杂交（FISH）检出t（11;14）亦可诊断MCL。

如果组织形态学特征和免疫表型符合典型MCL，但Cyclin D1和t（11;14）均阴性，则免疫组化检测SOX11，如果SOX11阳性，亦可诊断MCL，有条件单位可以加做FISH检测CCND2或CCND3重排。

（2）分型：MCL诊断后应进行分型：①经典型MCL，占MCL的绝大部分，生物学行为多样；②白血病性非淋巴结型MCL，多数临床呈惰性表现，评判可参考如下标准：a. 临床上惰性起病，白血病性表现，脾大而无淋巴结明显肿大；b. 生物学特点：不伴有复杂核型，免疫球蛋白重链可变区（IGHV）基因突变型，不表达或低表达SOX11，Ki-67％通常<10％。需要注意，少数白血病型非淋巴结性MCL侵袭性较高、容易出现迅速进展[10]–[12]。

原位套细胞肿瘤（in site mantle cell neoplasm, ISMCN）：指Cyclin D1阳性（常伴CCND1基因重排）的B细胞局限分布于淋巴滤泡套区，但未破坏淋巴结结构，并未达到MCL诊断标准。ISMCN常偶然被发现，很少出现进展，有时与其他淋巴瘤共存，可呈播散性表现。

（二）鉴别诊断

主要与其他B细胞淋巴增殖性疾病进行鉴别，尤其是慢性淋巴细胞白血病（CLL），具体请参考《B细胞慢性淋巴增殖性疾病诊断与鉴别诊断中国专家共识（2018 年版）》[13]。

（三）分期

经典型MCL按照Lugano修订的Ann Arbor 分期系统[14]分为Ⅰ期、Ⅱ期、Ⅱ期伴大肿块和Ⅲ期、Ⅳ期。白血病性非淋巴结型MCL尚无统一分期标准，可参考CLL相关标准（见《中国慢性淋巴细胞白血病/小淋巴细胞淋巴瘤的诊断与治疗指南》2018年版）[15]。

（四）预后

目前临床上普遍采用简易套细胞淋巴瘤国际预后评分系统（MIPI）和MIPI-c进行预后分层（表1、2）[16]–[17]。

**表1:** 简易套细胞淋巴瘤国际预后评分系统（MIPI）[16]

分数	年龄（岁）	ECOG评分（分）	LDH值/正常值	WBC（×10^9^/L）
0	<50	0～1	<0.67	<6.70
1	50～59		0.67～0.99	6.70～9.99
2	60～69	2～4	1.00～1.49	10.00～14.99
3	≥70		≥1.50	≥15.00

注：MIPI分组：低危组：0～3分；中危组：4～5分；高危组：6～11分。ECOG：美国东部肿瘤协作组；LDH：乳酸脱氢酶

**表2:** 结合Ki-67指数的联合MIPI预后评分系统（MIPI-c）[17]

MIPI-c分组	MIPI分组	Ki-67指数	患者比例（％）	5年总生存率（％）
低危	低危	<30％	32～44	85
低中危	低危	≥30％	5～9	72
	中危	<30％	25～29	
高中危	中危	≥30％	6～10	43
	高危	<30％	10～13	
高危	高危	≥30％	5～11	17

其他生物学预后指标包括：细胞遗传学异常如del（17p）或TP53突变、MYC扩增/易位、CDKN2A（9p）缺失等，Ki-67增殖指数，母细胞变型等[18]。有研究显示TP53突变、CDKN2A和TP53同时缺失的患者中位生存期不足2年[8],[19]–[21]，需要积极探索新的治疗方案。以上均为BTK抑制剂等新药时代前的预后因素，新药时代其预后意义不明。

三、治疗

（一）治疗指征

局限的ISMCN多不需要治疗，临床定期随诊。白血病性非淋巴结型MCL临床多表现为惰性病程，治疗指征可参考CLL，无症状且无治疗指征的患者可以先采取观察等待的策略，但应密切随访，中位至治疗时间2～3年。经典型MCL仅极少早期局限型患者可观察等待，绝大多数应在诊断后即开始治疗[22]。

（二）治疗前评估

治疗前（包括复发患者治疗前）应对患者进行全面评估，应至少包括：

1. 病史和体格检查（特别是浅表淋巴结和肝脾大小）。

2. 体能状态评分：ECOG评分。

3. B症状：发热、盗汗、体重减轻等。

4. 实验室检查：血常规，肝肾功能，血LDH、β_2_-微球蛋白。

5. HBV、HIV等病毒相关检测。

6. 病理检查：①淋巴结病理+免疫组化；②骨髓活检+免疫组化+流式细胞术分析免疫表型；③染色体核型分析和FISH技术检测t（11;14）。

7. 影像学检查：①推荐全身PET-CT检查或颈、胸、全腹部增强CT检查；②怀疑胃肠道受累时进行胃肠内镜检测，Ⅰ～Ⅱ期患者建议常规进行胃肠内镜检查；③母细胞型或考虑中枢神经系统受累时进行腰椎穿刺及磁共振成像（MRI）检查；④心脏彩超（左室射血分数）或多门控探测（MUGA）扫描：考虑应用蒽环类方案化疗时。

推荐进行IGHV突变检测以及FISH检测TP53缺失、CDNK2A缺失和MYC异常，有条件者可进行淋巴瘤相关的二代基因测序，包括TP53、ATM、CCND1、KMT2D、SP140、BIRC3、WHSC1、NOTCH1/2、SMARCA4、CARD11和UBR5等。

（三）一线治疗

1. Ann Arbor Ⅰ～Ⅱ期：对于少部分不伴高危因素的Ⅰ或连续型Ⅱ期（可放一个靶区放疗）患者，可采取类似于滤泡淋巴瘤的治疗策略，单纯受累野放疗（IRST）、免疫化疗联合或不联合IRST，放疗剂量30～36 Gy。而对于非连续型Ⅱ期且不伴高危因素患者，推荐进行常规免疫化疗（非强化方案）。治疗流程如图1。

**图1:**
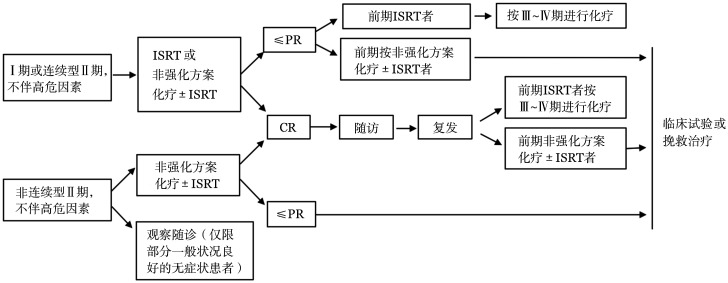
早期初治套细胞淋巴瘤治疗流程 注：ISRT：受累野放疗；CR：完全缓解；PR：部分缓解

对于伴有高危因素的Ⅰ～Ⅱ期患者，建议按照晚期（Ⅲ～Ⅳ期）进行治疗。高危因素包括：大肿块病变（≥5 cm）、Ki-67>30％、TP53突变/缺失、细胞形态为侵袭性变型等。

2. Ann Arbor Ⅲ～Ⅳ期或伴高危因素的Ⅰ～Ⅱ期：晚期MCL患者或有治疗指征的白血病性非淋巴结型MCL患者的治疗需要依据患者的年龄、一般状况和TP53等遗传学异常情况进行分层治疗，治疗选择流程如图2和表3。

**图2:**
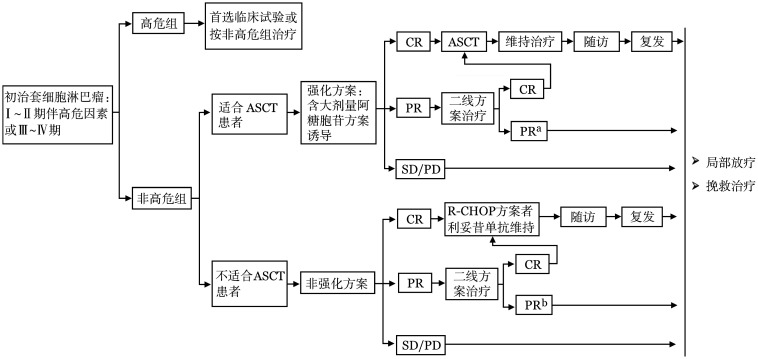
晚期初治套细胞淋巴瘤治疗流程 注：ASCT：自体造血干细胞移植；CR：完全缓解；PR：部分缓解；SD：疾病稳定；PD：疾病进展。^a^视具体方案决定是否需要更换后线治疗，较好的部分缓解患者可进行ASCT巩固；^b^视具体方案决定是否需要更换后线治疗

**表3:** 初治套细胞淋巴瘤治疗方案[5]

阶段	年轻、适合自体造血干细胞移植者（强化方案）		老年或不适合自体造血干细胞移植者（非强化方案）	
	治疗方案	参考用法	治疗方案	参考用法
诱导	①R-DHAP（奥沙利铂/卡铂/顺铂）[23]	R 375 mg/m^2^，每疗程第1天；Dex 40 mg/d，第1～4天；Ara-C 2.0 g/m^2^，每12 h 1次，第2天；铂类选择：顺铂100 mg/m^2^，持续输注24 h，第1天；或奥沙利铂130 mg或100 mg/m^2^，第1天；或卡铂AUC＝5 mg·ml^−1^·min^−1^	①B-R：苯达莫司汀+利妥昔单抗[24]	苯达莫司汀90 mg/m^2^，第2～3天；R 375 mg/m^2^，第1天，每4周为1个疗程
	②R-CHOP/R-DHAP交替[25]	CHOP：CTX 750 mg/m^2^，阿霉素50 mg/m^2^，VCR 1.4 mg/m^2^（最大2.0 mg），泼尼松100 mg，第1～5天； DHAP：Dex 40 mg/d，第1～4天；Ara-C 2.0 g/m^2^，每12 h 1次，第2天；顺铂100 mg/m^2^，持续输注24 h，第1天； R 375 mg/m^2^，每疗程第1天	②VR-CAP[26]	硼替佐米1.3 mg/m^2^，第1、4、8、11天；R 375 mg/m^2^，第1天；CTX 750 mg/m^2^，第1天；阿霉素50 mg/m^2^，第1天；泼尼松100 mg/m^2^，第1～5天
	③北欧方案：R-maxi-CHOP/R-HD-Ara-C交替[27]	CTX 1 200 mg/m^2^，阿霉素75 mg/m^2^，VCR 2 mg/d，泼尼松100 mg/d，第1～5天； Ara-C 2～3 g/m^2^，每12 h 1次×2 d；R 375 mg/m^2^，每疗程第1天	③R-CHOP	CTX 750 mg/m^2^，阿霉素50 mg/m^2^，VCR 1.4 mg/m^2^（最大2 mg），泼尼松100 mg/d第1～5天；R 375 mg/m^2^，每疗程第1天，停疗后每2～3个月维持1次，共2～3年
	④R-Hyper CVAD/R-MA方案交替[28]	Hyper-CVAD：CTX 300 mg/m^2^，每12 h 1次，第2～4天；VCR 1.4 mg/m^2^，第5、12天；Dex 40 mg/d，第2～5、12～15天； MA：MTX 1 000 mg/m^2^，第2天；Ara-C 3 g/m^2^，每12 h 1次，第3～4天； R 375 mg/ m^2^，第1天	④R^2^：利妥昔单抗+来那度胺[29]	诱导：来那度胺20 mg/d×21 d，28 d 1个疗程；R 375 mg/m^2^，第1、8、15、22天，第1疗程，以后每疗程1次，共9次； 维持：来那度胺15 mg/d×21 d，28 d为1个疗程；R 375 mg/m^2^，每2个疗程1次（8周）；至少36个疗程或至不能耐受/疾病进展
	⑤R-B/R-C序贯：利妥昔单抗+苯达莫司汀3个疗程后序贯 R+Ara-C 3个疗程[30]	R 375 mg/m^2^，第1天，苯达莫司汀90 mg/m^2^，第1～2天，每4周1个疗程；R 375 mg/m^2^，第1天，Ara-C 2～3 g/m^2^，每12 h 1次，第1～2天，每3周1个疗程	⑤BTK抑制剂+R方案[31]	R 375 mg/m^2^，第1个疗程，每周1次，第2～8个疗程，每疗程1次，每4周1个疗程，维持治疗期间每2个月1次，持续2年；BTK抑制剂根据说明持续应用至疾病进展
			⑥改良R-Hyper CVAD（年龄>65岁）[32]	R 375 mg/m^2^，第1天；CTX 300 mg/m^2^ 每12 h 1次，第1～3天；阿霉素25 mg/m^2^，持续输注，第1～2天；VCR 2 mg/d，静推，第3天；Dex 40 mg/d，第1～4天
			⑦R-BAC500[33]	R 375 mg/m^2^，第1天；苯达莫司汀70 mg/m^2^，第2～3天；Ara-C 500 mg/m^2^，第2～4天
巩固	自体造血干细胞移植			
维持	R或来那度胺	R 375 mg/m^2^，每2～3个月1次，通常维持2～3年[34]–[35]（1类）；来那度胺10～15 mg/d，第1～21天，每周期28 d，维持治疗24个月[36]		

注：R：利妥昔单抗；CTX：环磷酰胺；VCR：长春新碱；Dex：地塞米松；MTX：甲氨蝶呤；Ara-C：阿糖胞苷

对于年龄≤65岁且一般状况较好、适合自体造血干细胞移植（ASCT）的患者，应选择利妥昔单抗联合含中大剂量阿糖胞苷的方案诱导治疗，缓解后进行ASCT巩固。表3中的5个方案推荐等级相同，利妥昔单抗（R）-CHOP/R-DHAP序贯ASCT治疗方案是近年来临床研究采用较多的方案。铂类药物选择：LyMA研究显示，R-DHA+顺铂或卡铂疗效相当，而R-DHA+奥沙利铂无论是4年无进展生存（PFS）率（86.5％对65％，*P*＝0.02）还是总生存（OS）率（92％对74.9％，*P*＝0.03）均优于前两种铂类药物[23]。

对于年龄>65岁或一般状况较差、不适合ASCT的患者，则应选择不良反应较小、耐受性较好的方案进行联合化疗，联合利妥昔单抗化疗可提高患者的长期生存率。其中B-R、VR-CAP、R-CHOP和R^2^是优选方案（表3）。伊布替尼联合利妥昔单抗方案治疗初治老年非高增殖（Ki-67<50％）、肿瘤最大直径<10 cm和非母细胞变型MCL的有效率为96％［完全缓解（CR）率为71％］，3年PFS率为87％，OS率为94％[31]。有研究表明B-R方案较R-CHOP方案可显著延长PFS时间，且不良反应较小，但OS改善不显著[24]。

高危组包括TP53突变、TP53和CDNK2A缺失、侵袭性变型、MIPI-c高危组[18]。高危组患者常规治疗疗效差，目前没有标准治疗方案，利妥昔单抗联合中大剂量阿糖胞苷方案序贯ASCT虽然可在一定程度上延长患者的生存期，但总体预后较差，可积极探索以新药（如BTK抑制剂、BCL-2抑制剂、来那度胺）为基础的联合治疗、CAR-T细胞治疗和（或）异基因造血干细胞移植等。

3. 维持治疗：对于年轻患者，ASCT后予利妥昔单抗维持治疗（375 mg/m^2^，每2～3个月1次，共2～3年）可显著延长PFS时间和OS时间[34]–[35]。ASCT后予来那度胺10～15 mg/d，第1～21天，每周期28 d，维持治疗24个月，较不维持治疗者可显著延长PFS时间[36]。

对于年龄≥65岁的患者，R-CHOP方案治疗缓解后推荐予利妥昔单抗维持治疗（375 mg/m^2^，每2～3个月1次，维持2年或至疾病进展），可进一步改善OS[37]（1类）。对于接受B-R方案诱导化疗的老年初治患者，若达到CR，可不予利妥昔单抗维持，若仅达到部分缓解，利妥昔单抗维持可显著延长OS时间[38]。

（四）挽救治疗

挽救性治疗药物/方案见表4。一线治疗后复发患者首选参加设计良好的临床试验，无临床试验时依据患者是否具有高危因素进行选择，高危因素包括：TP53突变/缺失、CDKN2A缺失、侵袭性变型、Ki-67％>50％。

**表4:** 复发/难治套细胞淋巴瘤的挽救性治疗方案[22]

优选方案		其他方案
①	BTK抑制剂：	R-BAC500
	伊布替尼±利妥昔单抗	B-R
	泽布替尼	R-DHAP
	奥布替尼	R-GemOx
	阿可替尼	BTK抑制剂+R^2^（2B类）
②	R^2^（利妥昔单抗+来那度胺）	伊布替尼+维奈克拉 维奈克拉+来那度胺+利妥昔单抗（2B类） 维奈克拉±利妥昔单抗

非高危患者首选BTK抑制剂[39]–[42]或来那度胺+利妥昔单抗治疗，特别是24个月内复发患者，24个月后复发患者可首选以苯达莫司汀为主的联合化疗，如R-BAC或B-R方案[43]，或其他既往未使用的方案。诱导缓解后年轻、有条件患者行减低剂量预处理的异基因造血干细胞移植[44]，ASCT在复发/难治MCL患者中疗效欠佳，初诊治疗未应用ASCT，且二线治疗获得CR的患者可考虑。对于前期未应用利妥昔单抗维持治疗的患者，可在利妥昔单抗联合治疗有效后予利妥昔单抗维持治疗[45]。BTK抑制剂治疗后复发的患者，R-BAC方案有效率高达83％（CR率60％）[46]，是优选方案。

一线复发后伴高危因素或二线治疗未达CR或BTK抑制剂治疗失败患者应尽早考虑靶向CD19的CAR-T细胞治疗或异基因造血干细胞移植[47]。ZUMA-2试验[48]和TRANSCEND-NHL-001试验[49]表明，靶向CD19的CAR-T细胞治疗高危难治性MCL的有效率为84％～93％，CR率为59％～67％。

其他在MCL中有效的新药包括新一代非共价结合的BTK抑制剂如LOXO-305、PI3K抑制剂、BCL-2抑制剂、ROR1偶联单克隆抗体、抗CD20/CD3双克隆抗体等[47]，均处于临床研究阶段。

四、疗效评价

MCL的疗效评价标准参照Lugano 2014标准进行[14],[50]。有条件的患者首先考虑进行含PET-CT的疗效评价。治疗期间每2个疗程进行1次疗效评价，每4个疗程进行全面评价，包括PET-CT，直至CR。PET-CT阴性后不再进行此项检查，除非考虑疾病进展。由于MCL常侵犯外周血或骨髓，有条件单位可考虑进行微小残留病监测[51]。

五、随访

完成治疗后的前2年应每3个月进行一次随访，包括病史、查体、血细胞计数及生化检查，每6个月进行一次CT检查（包括颈部、胸部及全腹部）。完成治疗后第3～5年每半年进行一次随访，每12个月进行一次CT检查。5年后每年进行一次随访或有症状时进行检查，如有异常或考虑疾病进展，则进行增强CT检查等。

注：参照NCCN对证据和共识分类：1类：基于高水平证据，NCCN一致认为此项治疗合理；2A类：基于低水平证据，NCCN一致认为此项治疗合理；2B类：基于低水平证据，NCCN基本认为此项治疗合理；3类：基于任何水平证据，NCCN对此项治疗是否合理存在重大分歧。除非特殊说明，所有证据和共识均为2A类。
